# The adjuvant G3 promotes a Th1 polarizing innate immune response in equine PBMC

**DOI:** 10.1186/s13567-018-0602-2

**Published:** 2018-10-22

**Authors:** Stina Hellman, Bernt Hjertner, Bror Morein, Caroline Fossum

**Affiliations:** Department of Biomedical Sciences and Veterinary Public Health, Swedish University of Agricultural Sciences, SLU, Box 7028, 750 07 Uppsala, Sweden

## Abstract

The immunomodulatory effect of a new particulate adjuvant, G3, alone or in combination with agonists to TLR2/1 or TLR5 was evaluated in cultures of equine PBMC. Exposure to the G3 adjuvant up-regulated genes encoding IFN-γ, IL-1β, IL-6, IL-8, IL-12p40 and IL-23p19 in the majority of the horses tested, indicating that the G3 adjuvant induced a pro-inflammatory and Th1 dominated profile. In accordance, genes encoding IL-13, IL-4, IL-10 and TGF-β remained unaffected and genes encoding IFN-α, IL-17A and TNF-α were only occasionally and weakly induced. The two TLR agonists Pam3CSK4 (TLR2/1) and FliC (TLR5) induced cytokine profiles characterized by a clear induction of IL-10 as well as up-regulation of the genes encoding IL-1β, IL-6 and IL-8. The presence of G3 modified this response, in particular by reducing the FliC and Pam3CSK4 induced production of IL-10. Furthermore, G3 acted in synergy with Pam3CSK4 in enhancing the production of IFN-γ whereas G3 combined with FliC increased the gene expression of IL-8. Thus, the G3 adjuvant seems to have the capacity to promote a Th1 polarizing innate immune response in eqPBMC, both by favouring IFN-γ production and by reducing production of IL-10 induced by co-delivered molecules. These features make G3 an interesting candidate to further evaluate for its potential as an adjuvant in equine vaccines.

**Electronic supplementary material:**

The online version of this article (10.1186/s13567-018-0602-2) contains supplementary material, which is available to authorized users.

## Introduction

A variety of adjuvants including aluminium salts, emulsions, carbomers and immune stimulatory complexes (ISCOMs) are today used in animal vaccines [1]. Based on their physical form and mode of action, these adjuvants have been categorized as particulate formulations, immunomodulatory molecules or a combination thereof. Particulate formulations primarily enhance uptake by antigen presenting cells [2, 3] but may also modulate innate immune responses [4–6]. This effect can be further improved by inclusion of other immunomodulatory molecules in the vaccine formula. Accordingly, compounds such as Toll-like receptor (TLR) agonists, polyphosphazenes and cytokines are currently under investigation as additives in vaccines [1, 7, 8]. The best-known example of such an adjuvant complex is AS04 based on aluminium salts combined with the TLR4 agonist monophosphoryl lipid A [9]. The strategy to combine adjuvant components in order to improve vaccine efficacy has also been examined for veterinary use, e.g. in vaccines for cattle, pigs and poultry [1].

In the present study, a novel adjuvant “G3” was evaluated in cultures of equine peripheral blood mononuclear cells (eqPBMC). The G3 adjuvant is a 20 nm particle formulated of cholesterol and QuilA components extracted from the tree *Quillaja saponaria Molina* that induce potent antibody and T cell responses to H1N1 influenza virus (Patent no. WO 2013/05/1994). Subsequent evaluations [10] demonstrate that G3 with an incorporated diterpene enhances immune protection to H1N1 influenza virus in mice challenged with a strain antigenically distinct from that used for immunisation. This protection was based on cytotoxic T lymphocytes targeting the nucleoprotein and polymerase A. A similar Th1 polarization was indicated by induction of IFN-γ/IL-2 double producing cells as determined by FluoroSpot and production of IgG2a in mice immunized with G3 adjuvanted influenza antigens [11]. In vitro, G3 alone induced IFN-γ production as well as increased the expression of maturation markers in cultures of human monocyte-derived dendritic cells [10].

The present study evaluated effects by G3 on eqPBMC stand alone or in combination with the TLR2/1 agonist Pam3CSK4 or the TLR5 agonist FliC. Both the recombinant flagellin protein FliC and the synthetic triacylated lipoprotein Pam3CSK4 have been extensively studied in several species and successfully included in several vaccine constructs [reviewed in 12, 13], but only limited data are available on their effects in the horse [14–17]. We were therefore motivated to study cytokine profiles of eqPBMC cultured in the presence of these two compounds stand alone or in combination with G3. As read out, transcription of cytokine genes was measured by qPCR and the production of IFN-γ and IL-10 was verified by ELISA.

## Materials and methods

### Isolation of eqPBMC

Healthy horses housed at the Department of Clinical Sciences, SLU, Uppsala, Sweden were used in the study. These horses (Swedish Warmblood, geldings and mares, age 9–14 years) are clinically examined including complete blood counts, and vaccinated for tetanus and influenza on a regular basis. The sampling was approved by the Ethical Committee for Animal Experiments in Uppsala. Blood was collected in heparinized tubes from the jugular vein. After 15–20 min sedimentation, blood plasma was collected and eqPBMC were isolated by centrifugation on Ficoll Paque (Amersham Pharmacia Biotech, Uppsala, Sweden). Cells were washed three times in PBS and suspended in growth medium, i.e. RPMI 1640 medium (BioWhittaker, Cambrex Bioscience, Verviers, Belgium) supplemented with HEPES (20 mM), l-glutamine (2 mM), penicillin (200 IU/mL), streptomycin (100 µg/mL), 2-mercaptoethanol (50 µM), and 5% fetal calf serum (Invitrogen, Life Technologies, Carlsbad, CA, USA).

### Culture conditions for gene expression analysis

Between 5 and 10 × 10^6^ eqPBMC in one mL medium were seeded in 6-well plates (Nunclon; Nunc, Roskilde, Denmark), incubated for 30 min at 37 °C in 7% CO_2_, whereafter one mL of the adjuvant “G3” (MoreinX AB, Uppsala, Sweden) at final concentrations 1, 3 or 5 µg/mL was added to the cultures. PBMCs were also cultured in the presence of either FliC (0.1 µg/mL; VacciGrade™, InvivoGen, San Diego, CA, USA), Pam3CSK4 (0.5 µg/mL; InvivoGen) or G3 (3 µg/mL), or in combinations thereof. The combinations of G3 with FliC or Pam3CSK4 were pre-incubated for 15 min in growth medium before being added to the cultures. After 18 h, cells were harvested for gene transcription analysis. As controls, freshly isolated eqPBMC or eqPBMC cultured in plain growth medium for 18 h were used.

### RNA extraction and cDNA synthesis

RNA extraction and cDNA synthesis were performed as previously described [18]. In brief, RNA was extracted by combining Trizol (Invitrogen, Carlsbad, CA, USA) with the column-based E.Z.N.A. total RNA kit (Omega Biotek, Norcross, GA, USA). RNA quantity and purity was estimated by spectrophotometry (NanoDrop ND-1000, NanoDrop Technologies, Montchanin, DE, USA) and RNA quality index (RQI) was estimated to ≥ 9.8 using capillary gel electrophoresis (Experion RNA StdSense Analysis Kit, Bio-Rad Laboratories, Solna, Sweden). After treating 0.4–1 μg of RNA with RQ1 RNAse-free DNAse (Promega, Madison, WI, USA) cDNA was synthesized (GoScript Reverse transcription system; Promega) and diluted 5 × before storage at −20 °C.

### qPCR

Published primer pairs for genes encoding equine IFN-α, IFN-γ, IL-1β, IL-4, IL-6, IL-8, IL-10, IL-12p40, IL-13, IL-17A, IL-23p19, TGF-β, and TNF-α were used under conditions optimised in house (Additional file 1). Duplicate reactions of 2 µL cDNA in 23 µL Quantitect SYBR Green PCR mix (Qiagen) were run in an IQ5 or a CFX96 Touch PCR machine (Bio-Rad) with an initial cycle of 95 °C for 15 min followed by 40 cycles of 95 °C for 15 s, the assay specific annealing temperature for 30 s and 72 °C for 30 s. Five reference genes; β2M, HPRT, RPL32, SDHA and TFRC [18], were evaluated for their expression stability in eqPBMC stimulated or not with G3 using the geNorm software (qBase^PLUS^, Biogazelle). The genes were scored based on a gene stability parameter (*M*) and a coefficient of variation (C*V*), where *M* values < 0.5 and *CV* values < 0.2 indicate high expression stability. The genes RPL32 (M = 0.339; CV = 0.128) and SDHA (M = 0.329; CV = 0.117) were accordingly selected for normalization prior to calibration of stimulated samples to their medium controls [19]. Genes reaching fold change (FC) values < 0.5 or > 2 were considered as differentially expressed.

### Culture conditions for analysis of IFN-γ and IL-10 production

EqPBMC (4 × 10^5^ in 100 µL medium) were incubated for 30 min in 96-well round bottom plates (Nunc) before addition of 100 µL growth medium containing G3 (final concentration: 3 or 5 µg/mL), FliC (0.1 µg/mL), Pam3CSK4 (0.5 µg/mL) or G3 mixed with FliC or Pam3CSK4. As controls, eqPBMC were cultured in plain growth medium or in the presence of phorbol 12-myristate 13-acetate (PMA; 0.05 µg/mL; Sigma-Aldrich^®^) and Ionomycin (1 µg/mL; Sigma-Aldrich^®^). Cell supernatants were collected after 48 h and stored at −20 °C until analysed for the presence of IFN-γ or IL-10 using commercial ELISA kits (Equine IFN-γ development kit, Mabtech, Nacka strand, Sweden; Equine IL-10 ELISA Kit, Thermo Scientific, Frederick, MD, USA) according to the manufacturer’s instructions.

### Kinetic studies

EqPBMC (2.5–5 × 10^6^ cells/mL) were seeded in 6-well plates and stimulated with G3 (5 µg/mL) and Pam3CSK4 (0.5 µg/mL) added simultaneously or sequentially (6 or 12 h apart). Cells were harvested 18 h after the first stimuli and analysed for IFN-γ mRNA expression. In addition, eqPBMC (4 × 10^6^ cells/mL) were seeded in 96-well round bottom plates and stimulated with G3 and Pam3CSK4 added simultaneously or sequentially after 2, 4, 6 or 8 h. In parallel, G3 or Pam3CSK4 were added alone after 0, 2, 4, 6 and 8 h of culture. All supernatants were collected after a total culture time of 48 h and stored at −20 °C until analysed for the presence of IFN-γ.

### Data analysis

Statistical analysis was performed using the software Prism 5.0 (Graphpad Software, Inc., CA, USA). Normal distribution of data was confirmed using the Kolmogorov–Smirnov test. Differences between treatments were determined using the repeated measures ANOVA followed by Tukey’s multiple comparison test where *P*-values < 0.05 were regarded as significant. When indicated, variability of gene expression data is reported as mean ± SD.

## Results

### Transcriptional response to G3

**Figure 1:**
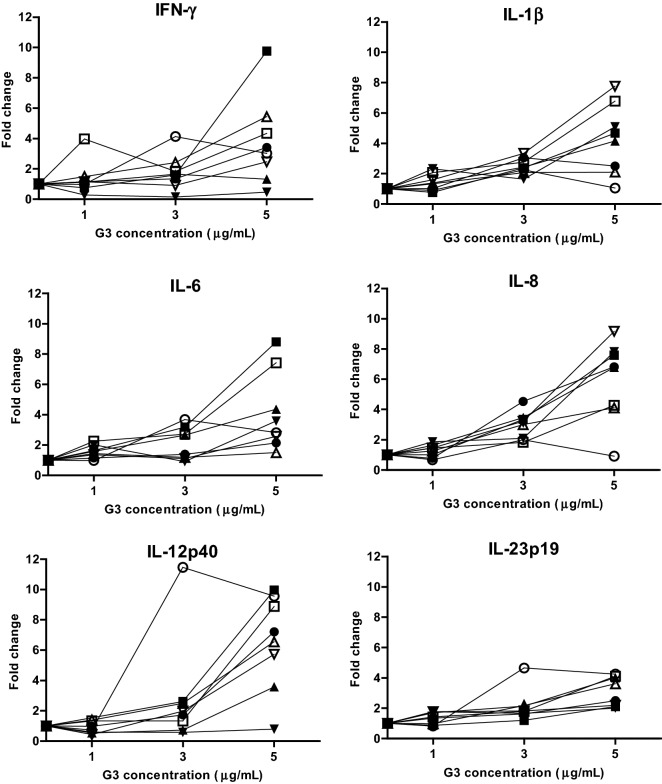
**Relative expression of the genes encoding IFN-γ, IL-1β, IL-6, IL-8, IL-12p40 and IL-23p19 in eqPBMC.** PBMC collected from eight horses were cultured in various concentrations of G3 (1, 3 or 5 µg/mL) or in plain growth medium for 18 h. The cytokine gene expression was normalized to the geometric mean for the reference genes (SDHA and RPL32) and calibrated to that in the medium control.

EqPBMC collected from eight horses were cultured for 18 h in the presence of G3 (1, 3 or 5 µg/mL) followed by expression analysis of 13 immune-related genes. In general, there was a variation in gene-expression levels between horses being especially evident at the concentration of 5 µg G3 per mL (Figure 1). At this concentration, the genes encoding IFN-γ, IL-1β, IL-6, IL-8, IL-12p40 and IL-23p19 were up-regulated in PBMC from six or more of the eight horses. Increased expression of the genes for IL-13 and IL-17A was observed in two (FC: 2.6 and 3.1) and three horses (FC: 3.6, 4.3 and 4.9), respectively. Exposure to a lower concentration of G3 (3 µg/mL) up-regulated the genes encoding IL-1β (in 7/8 horses), IL-6 (in 4/8 horses) and IL-8 (in 7/8 horses) but with lower FC values. Only one horse displayed the highest expression at 3 µg G3 per mL for the genes encoding IFN-γ, IL-1β, IL-6, IL-8, IL-12p40 and IL-23p19. The genes encoding IFN-α, IL-4, IL-10, TGF-β and TNF-α were not differentially expressed at any of the G3 concentrations tested (data not shown).

### Transcriptional response to G3 and TLR agonists

**Figure 2:**
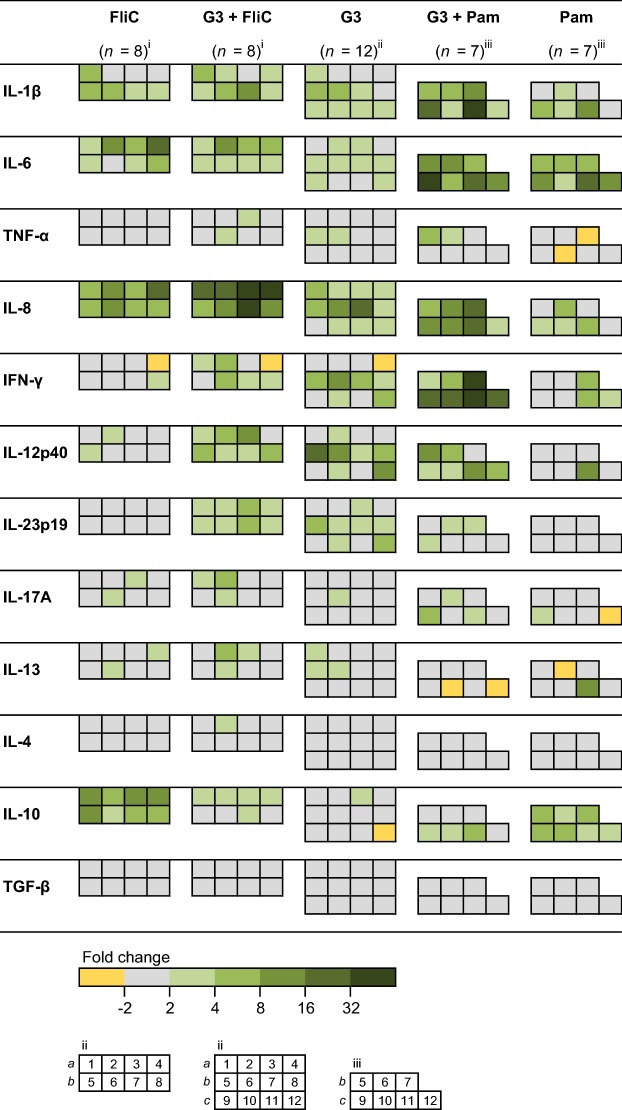
**Relative expression of cytokine genes in eqPBMC incubated in the presence of G3, FliC or Pam3CSK4, alone or in combination.** The PBMC were collected from twelve horses and cultured in the presence of G3 (*n* = 12), FliC (*n* = 8), Pam3CSK4 (*n* = 7), G3 + FliC (*n* = 8) and G3 + Pam3CSK4 (*n* = 7). The horses (nos. 1–12) were sampled four at a time at three occasions (a, b and c). Results from each horse are shown as indicated in the footnote to the heat map (i, ii, and iii). The cytokine gene expression was normalized to the geometric mean for the reference genes (SDHA and RPL32) and calibrated to that in the medium control. Five shades of green colour represent up-regulation ranging between FC > 2 (the brightest green) and FC > 32 (the darkest green). Grey and yellow colour represents no differential expression and down-regulation (FC < 0.5), respectively.

**Figure 3:**
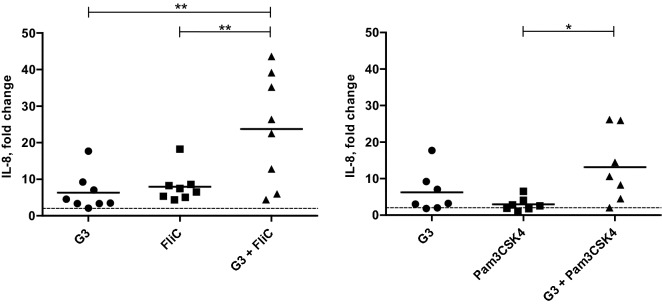
**Relative expression of the gene encoding IL-8 in eqPBMC after 18** **h of incubation in the presence of G3, FliC, Pam3CSK or combinations thereof.** The cytokine gene expression was normalized to the geometric mean for the reference genes (SDHA and RPL32) and calibrated to that in the medium control. FC > 2 (indicated by dashed line) were considered as up-regulated. *P < 0.05, **P < 0.01.

**Figure 4:**
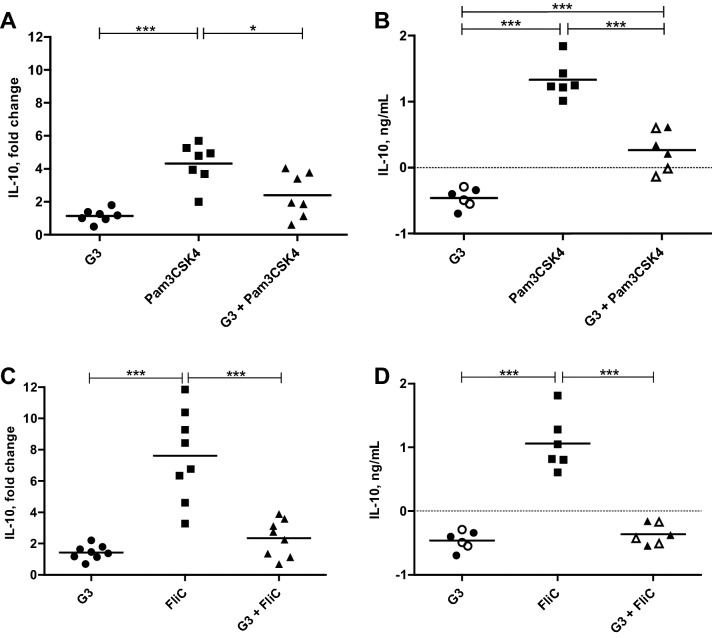
**Relative gene expression (A, C**) **and protein levels (B, D) of IL-10 in eqPBMC cultured for 18 or 48** **h in the presence of G3, FliC, Pam3CSK4 or combinations thereof.** The cytokine gene expression was normalized to the geometric mean for the reference genes (SDHA and RPL32) and calibrated to that in the medium control. The protein expression data are presented as the estimated IL-10 levels after subtraction of the spontaneous IL-10 production in parallel cultures. Experiments for gene expression and protein analyses were conducted independently. Closed and open symbols indicate 3 µg G3/mL and 5 µg G3/mL, respectively. *P < 0.05 and ***P < 0.001.

To study the effect of G3 in combination with the other immunostimulatory molecules, eqPBMC were cultured with G3 (3 µg/mL) alone or in combination with Pam3CSK4 or FliC (Figure 2). Alone, these TLR agonists up-regulated IL-1β, IL-6, IL-8 and IL-10. In addition, Pam3CSK4 up-regulated IFN-γ in PBMC from three out of seven horses (FC: 5.2, 5.5 and 5.5). As observed in the previous experiments, G3 alone induced expression of IL-1β, IL-6 and IL-8 in ≥ 8/12 horses and IFN-γ and IL-12p40 in 6/12 horses, but not IL-10. Gene expression of IL-1β, IL-6 and IL-8 was further increased when G3 was combined with Pam3CSK4 or FliC (Figure 2), being most evident for the IL-8 expression induced by the combination of G3 with FliC (*P* < 0.01; Figure 3). None of the combinations affected the gene expression of IL-12p40 or IL-23p19 (Figure 2) whereas the presence of G3 significantly decreased the gene expression of IL-10 induced by Pam3CSK4 (*P* < 0.05; Figure 4A) or FliC (*P* < 0.001; Figure 4C).

### G3 inhibits IL-10 production

The inhibition of IL-10 gene transcription was also reflected at the protein level in eqPBMC after incubation for 48 h in the presence of G3 and Pam3CSK4 (Figure 4B) or FliC (Figure 4D). After subtraction of the spontaneous production of IL-10 recorded in control cultures (range 0.7–1.2 ng IL-10/mL medium), the levels of IL-10 in supernatants from eqPBMC exposed to PMA/ionomycin ranged from 8.6 to 12.2 ng/mL. FliC and Pam3CSK4 alone induced concentrations between 0.6 and 1.3 and 1.0–1.8 ng IL-10/mL, respectively, which were significantly reduced (*P* < 0.001) by the presence of G3.

### G3 combined with Pam3CSK4 enhance production of IFN-γ

**Figure 5:**
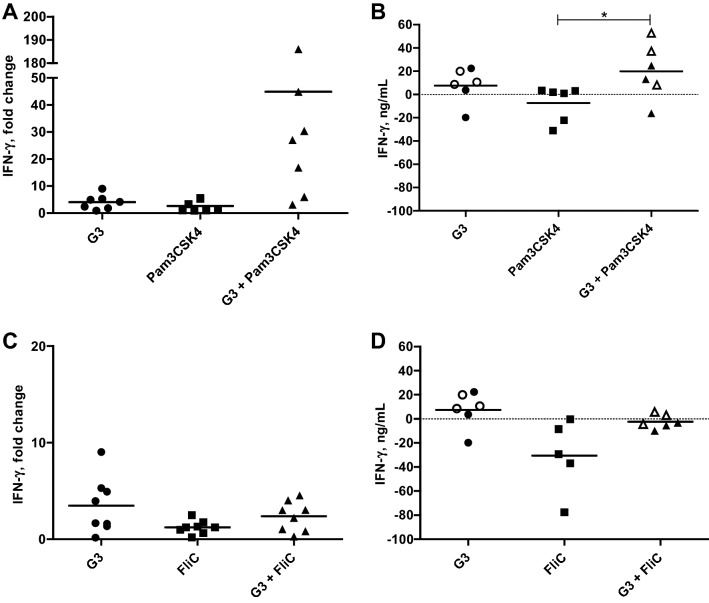
**Relative gene expression (A, C) and protein levels (B, D) of IFN-γ in eqPBMC cultured for 18 or 48** **h in the presence of G3, Pam3CSK4, FliC and combinations thereof.** The cytokine gene expression was normalized to the geometric mean for the reference genes (SDHA and RPL32) and calibrated to that in the medium control. The protein expression data are presented as the estimated IFN-γ levels after subtraction of the spontaneous IFN-γ production in parallel control cultures. Experiments for gene expression and protein analyses were conducted independently. Closed and open symbols indicate 3 µg G3/mL and 5 µg G3/mL, respectively. *P < 0.05.

Dual exposure to G3 and Pam3CSK4 markedly increased the gene expression of IFN-γ. This effect was observed in PBMC from five out of seven horses, with 7–187 times higher IFN-γ gene expression compared to when exposed to G3 or Pam3CSK4 alone (Figure 5A). These effects were confirmed by the protein levels of IFN-γ recorded in cell culture supernatants (Figure 5B). After subtraction of the spontaneous IFN-γ production (range 0.7–42.8 ng IFN-γ/mL), eqPBMC stimulated with PMA and ionomycin produced between 34 and 420 ng IFN-γ/mL whereas G3 alone induced 3–22 ng IFN-γ/mL. In accordance with the gene expression data, the IFN-γ production increased when G3 was combined with Pam3CSK4, ranging from 8 to 52 ng IFN-γ/mL (Figure 5B) but not when combined with FliC (Figures 5C and D).

### Effects of order and time elapse between addition of G3 and Pam3CSK4 on IFN-γ production

**Figure 6:**
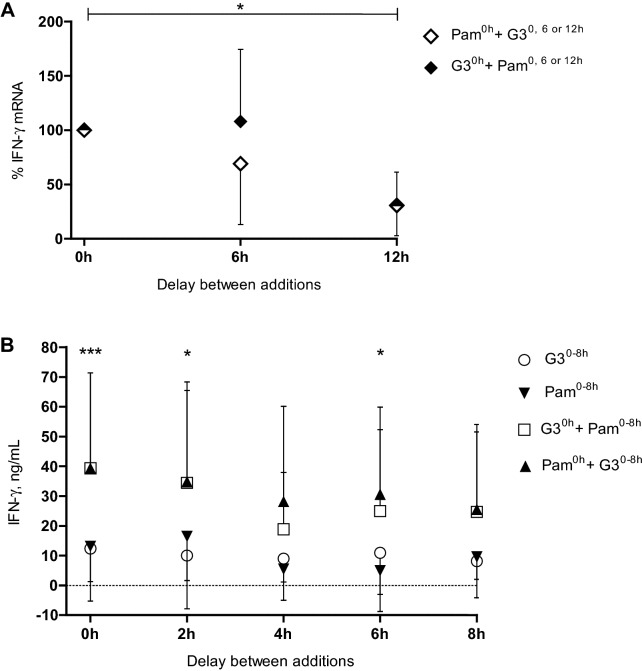
**The combined effect of G3 and Pam3CSK4 on IFN-γ production by eqPBMC when the two inducers were added simultaneously or sequentially. A** The IFN-γ gene expression (FC values) obtained for sequentially stimulated cells were for each horse transformed to percentages of that recorded at simultaneous addition of G3 and Pam3CSK4. In the figure, FC values of simultaneously induced eqPBMC are displayed as 100%. Closed symbols represent cultures where G3 was added as first inducer (0 h) and Pam3CSK4 as second inducer (6 or 12 h). Open symbols represent cultures where Pam3CSK4 was added as first inducer (0 h) and G3 as second inducer (6 or 12 h). Total culture time was 18 h. Results are given as mean values ± SD, *n* = 6. **B** Protein concentrations of IFN-γ (ng/mL) in supernatants from eqPBMC cultured in the presence of G3 and Pam3CSK4 added simultaneously (0 h + 0 h), sequentially (0 h + 2, 4, 6 or 8 h) or separately (0, 2, 4, 6 or 8 h). Inverted pyramids = Pam3CSK4 alone; circles = G3 alone; squares = G3 followed by Pam3CSK4; pyramids = Pam3CSK4 followed by G3. For statistical analysis, the IFN-γ concentrations recorded in cultures exposed to Pam3CSK4 or G3 alone were pooled and compared with pooled values from cultures stimulated by the combinations of G3 and Pam3CSK4. Total culture time was 48 h. Results are given as mean values ± SD, n = 6. *P < 0.05 and ***P < 0.001.

To further elucidate the combined effect of G3 and Pam3CSK4 on the IFN-γ induction, consecutive order and time elapsing between additions of the two inducers were studied. In accordance with the previous data, simultaneous exposure to G3 and Pam3CSK4 increased the gene expression of IFN-γ nine to 71 times compared to that induced by G3 or Pam3CSK4 alone. To compensate for the individual variation, FC values of sequentially stimulated eqPBMC were related to FC values recorded at simultaneous stimulation with G3 and Pam3CSK4 which was set to 100%. The enhancing effect was retained when G3 and Pam3CSK4 were added within 6 h, regardless of the order of addition. However, the gene expression of IFN-γ was significantly decreased (*P* < 0.05) when G3 or Pam3CSK4 were added twelve hours apart (Figure 6A).

To study this effect in further detail at the protein level, IFN-γ induced by G3 and Pam3CSK4 added within closer intervals were at each occasion (2, 4, 6 and 8 h) compared to that in parallel cultures stimulated by G3 or Pam3CSK4 alone (Figure 6B). In line with the transcript analysis, the order of addition was not pivotal for the IFN-γ increase and simultaneous addition of G3 and Pam3CSK4 generated significantly higher (*P* < 0.001) concentrations of IFN-γ compared to that induced by G3 or Pam3CSK4 alone. The enhancing effect remained when G3 and Pam3CSK4 were added up to 6 h apart (*P* < 0.05).

## Discussion

The present study was conducted to explore the possibility to modulate immune reactions using the G3 adjuvant stand alone or in combination with Pam3CSK4 or FliC. The magnitude of the responses to G3, FliC or Pam3CSK4 varied between PBMC collected from different horses. Therefore, the responses to the various cytokine inducers, or combinations of those, were compared within each individual. Inter-individual variations in cytokine responses are found in most mammalian species, including the horse [20–23], which can be explained by genetic as well as environmental factors. Despite these variations, distinct patterns of gene regulation was revealed at stimulation with G3 or any of the two TLR agonists. Thus, it can be assumed that the large variation recorded for the IFN-γ and IL-8 responses in the present study, reflects that of an out-bred population of vaccine recipients.

Both Pam3CSK4 and FliC tended to up-regulate the genes encoding IL-1β, IL-6, IL-8 and IL-10. Previous studies conducted in the horse using flagellin reveal an increased expression of the genes for IL-6 and IL-8 in primary cultures of equine keratinocytes [17] as well as for TNF-α and IL-10 in equine neutrophils [16]. Thus, flagellin/FliC seems to induce similar cytokine responses in equine cells as in other species [24]. Likewise, Pam3CSK4 induces equine monocytes to produce IL-1β, IL-6, IL-10 and TNF-α [14] as it does in human and murine cells [25, 26]. In the present study, Pam3CSK4 did not induce gene expression of TNF-α, possibly explained by a spontaneous increase in gene expression of TNF-α seen after 18 h of incubation (data not shown) being congruent with a high spontaneous release of TNF-α found in supernatants of unstimulated eqPBMC [22]. Also, a spontaneous induction of IL-1β was recorded in the control cultures. However, the gene expression of IL-1β increased further by the presence of Pam3CSK4, in agreement with previous observations where stimulation with Pam3CSK4 induced higher levels of IL-1β compared to TNF-α in cultures of equine monocytes [14]. Furthermore, the expression of TNF-α is almost instant and was not detectable after 2 h culture of equine whole blood exposed to LPS [27]. Thus, it cannot be excluded that G3 induced a rapid transient production of TNF-α which was not detected in the present experimental set-up. Regardless, FliC and Pam3CSK4 are useful tools to delineate cytokine responses in cultures of eqPBMC.

The increase of IL-8 and IFN-γ in response to G3 combined with FliC or Pam3CSK4 was more than additive, indicating synergy. Stimulation of multiple pattern recognition receptors (PRRs) are considered important for tailoring the innate cytokine production [28]. Indeed, several studies report synergistic activation of innate and adaptive immune responses by combining multiple TLR agonists in a vaccine [7, 29–32]. Considering that most pathogens contain multiple PAMPs, activation of more than one receptor is probably important for the host to mount effective immune responses to infections. These results are in line with earlier studies [10] showing that G3 alone induced CD8^+^ T cells as well as antibodies specific for influenza virus antigens in mice, but protection against challenge with a heterologous influenza virus strain was only achieved when G3 was formulated with an additional immunostimulant, i.e., a steviol glycoside [33].

Several studies report preserved synergy effects by sequential stimulation of PRRs [34–36]. Therefore, the kinetics behind the synergy effects seen on IFN-γ production was studied by adding G3 and Pam3CSK4 to eqPBMC in different order and at different time points. Enhanced production of IFN-γ was retained when G3 and Pam3CSK4 were added within a 2–6 h time window, indicating that the synergy on IFN-γ production was due to an indirect rather than a direct interaction between G3 and Pam3CSK4. The synergy effect was also independent of the order of addition, suggesting that both inducers may have “priming” capacities [37]. The enhancement of IFN-γ production can possibly explain why the presence of G3 reduced the production of IL-10 induced by both TLR agonists. However, the modes of action behind synergistic and negative cross-talks between PRRs are largely unclear and is probably affected by a range of factors [38].

The immunomodulatory component of G3 is the saponin Quil A, here formulated into nanoparticles with cholesterol. A proposed mechanisms involved in the adjuvant activity of Quil A was recently reviewed [39], suggesting that T cells could be directly activated by saponin aldehyde side chains interacting with amino acid residues present in the TCRs. Another possible interaction is through sugar moieties binding to lectin receptors on immune cells [40, 41]. Thus, it is likely that G3 physically interacts with cellular receptors and starts a signalling cascade that can be further modified by inclusion of other immunomodulatory compounds. Taken together, the adjuvant G3 seems to polarize the immune response by inducing a cytokine profile dominated by pro-inflammatory and Th1-associated cytokines. This profiling is highly desired for vaccines against pathogens where immune protection largely depends on cell-mediated immunity. Therefore, these results make it interesting to further evaluate the potential of G3 as an adjuvant in equine vaccines.

## Additional file

**Additional file 1.** **Primer details and optimized qPCR conditions**. ^a^PCR efficiency estimated on serial dilutions of reference cDNA.
