# Supplementary material for: The adjuvant G3 promotes a Th1 polarizing innate immune response in equine PBMC
Source: Vet Res. 2018 Oct 22;49:108. doi: 10.1186/s13567-018-0602-2 (PMC6389152; doi:10.1186/s13567-018-0602-2)
Supplement: Supplementary file 1 — Additional file 1. Primer details and optimized qPCR conditions. aPCR efficiency estimated on serial dilutions of reference cDNA. [file 13567_2018_602_MOESM1_ESM.docx]

| Target  gene | Primer sequence | Primer location | Target sequence | Anneal temp (°C) | Primer conc (nM) | Eff  (%)^a^ | r^2^ | Melt point (°C) |
| --- | --- | --- | --- | --- | --- | --- | --- | --- |
| IFN-α  (18) | F: CCAGTTCCGGAAGCCTCAAG | 207–226 | NM_001099441.1 | 55 | 300 | 102 | 0.996 | 79 |
|  | R: GAAGAGGTGGAAGATCTGTTGGAT | 207–226 |  |  |  |  |  |  |
| IFN-γ  (42) | F: CCAGCGCAAAGCAATAAGTG | 448-467 | NM_001081949.1 | 55 | 400 | 101 | 0.999 | 78 |
|  | R: GGCCTCGAAACGGATTCTG | 548-530 |  |  |  |  |  |  |
| IL-1β  (43) | F: ACCATAAATCCCTGGTGCTG | 447-466 | NM_001082526.1 | 55 | 400 | 96 | 0.998 | 80.5 |
|  | R: CATCCCACAAGACAGGTACA | 625-606 |  |  |  |  |  |  |
| IL-4  (44) | F: CAAAACGCTGAACAACCTCA | 105-124 | NM_001082519.1 | 55 | 400 | 98 | 0.997 | 80.5 |
|  | R: CTGTTGAAGCACCTTTGCAG | 353-331 |  |  |  |  |  |  |
| IL-6  (45) | F: GAAAAAGACGGATGCTTCCAATCTG | 280-304 | NM_001082496.2 | 55 | 400 | 97 | 0.997 | 76.5 |
|  | R: TCCGAAAGACCAGTGGTGATTTT | 353-331 |  |  |  |  |  |  |
| IL-8  (46) | F: TTGGCCGTCTTCCTGCTTT | 120-138 | NM_001083951.2 | 55 | 400 | 101 | 1.000 | 80.5 |
|  | R: GGTTTGGAGTGCGTCTTGATG | 220-200 |  |  |  |  |  |  |
| IL-10  (44) | F: GTCATCGATTTCTGCCCTGT | 377-396 | NM_001082490.1 | 58 | 500 | 97.5 | 0.996 | 78.5 |
|  | R: GCTTCGTTCCCTAGGATGC | 557-539 |  |  |  |  |  |  |
| IL-12p40 (18) | F: TGCTGTTCACAAGCTCAAGTATGA | 642-665 | NM_001082516.1 | 59 | 300 | 101 | 0.998 | 78 |
|  | R: GGGTGGGTCTGGTTTGATGA | 717-698 |  |  |  |  |  |  |
| IL-13  (47) | F: GTGGAGCGTCAACCTGACA | 156-174 | NM_001143791.1 | 56 | 400 | 97.5 | 0.994 | 81.5 |
|  | R: CTTCCGCGTGTTTTGGAT | 249-232 |  |  |  |  |  |  |
| IL-17A  (18) | F: CCAGAAGGGCCTCAGATTACCACA | 173-196 | NM_001143792.1 | 56 | 300 | 95 | 0.996 | 84 |
|  | R: ACCTTCCCTTCGGCATTGACACAG | 311-288 |  |  |  |  |  |  |
| IL-23p19  (18) | F: AGTGCGAGGATGGCTGTGAT | 291-310 | NM_001082522.2 | 59 | 400 | 97 | 0.992 | 82 |
|  | R: GGCTCCCCTGTGAAAATGTCT | 414-394 |  |  |  |  |  |  |
| TGF-β  (47) | F: TGACAGCAAAGATAACACACTCC | 679-711 | NM_001081849.1 | 55 | 400 | 94.5 | 0.999 | 81.5 |
|  | R: TCAATGGTGGCCAGATCA | 766-749 |  |  |  |  |  |  |
| TNF-α  (45) | F: GCTCCAGACGGTGCTTGTG | 112-130 | NM_001081819.2 | 56 | 500 | 100 | 0.998 | 82.5 |
|  | R: GCCGATCACCCCAAAGTG | 206-189 |  |  |  |  |  |  |

[18] Hjertner B, Olofsson KM, Lindberg R, Fuxler L, Fossum C (2013) Expression of reference genes and T helper 17 associated cytokine genes in the equine intestinal tract. Vet J 197:817–23

[42] Figueiredo MD, Salter CE, Andrietti ALP, Vandenplas ML, Hurley DJ, Moore JN (2009) Validation of a reliable set of primer pairs for measuring gene expression by real-time quantitative RT-PCR in equine leukocytes. Vet Immunol Immunopathol 131:65–72

[43] Beekman L, Tohver T, Léguillette R (2012) Comparison of cytokine mRNA expression in the bronchoalveolar lavage fluid of horses with inflammatory airway disease and bronchoalveolar lavage mastocytosis or neutrophilia using REST software analysis. J Vet Intern Med 26:153–61

[44] Sánchez-Matamoros A, Kukielka D, De las Heras AI, Sánchez-Vizcaíno JM (2013) Development and evaluation of a SYBR Green real-time RT-PCR assay for evaluation of cytokine gene expression in horse. Cytokine 61:50–3

[45] Liu T, Nerren J, Liu M, Martens R, Cohen N (2009) Basal and stimulus-induced cytokine expression is selectively impaired in peripheral blood mononuclear cells of newborn foals. Vaccine 27:674–83

[46] Lopes MAF, Salter CE, Vandenplas ML, Berghaus R, Hurley DJ, Moore JN (2010) Expression of inflammation-associated genes in circulating leukocytes collected from horses with gastrointestinal tract disease. Am J Vet Res 71:915–24

[47] Padoan E, Ferraresso S, Pegolo S, Castagnaro M, Barnini C, Bargelloni L (2013) Real time RT-PCR analysis of inflammatory mediator expression in recurrent airway obstruction-affected horses. Vet Immunol Immunopathol 156:190–9
